# Acetylshikonin mitigates diet-induced MASLD by targeting PPARγ-mediated metabolic dysfunction

**DOI:** 10.3389/fphar.2026.1735481

**Published:** 2026-02-23

**Authors:** Ling Ou, Qian Du, Jiayang Liu, Haiyan Tai, Yinghan Chai, Xiaoqiong Tan, Bing Li, Lirong Tan, Ying Cao, Tingting Zhu

**Affiliations:** 1 GuiZhou University Medical College, Guiyang, Guizhou, China; 2 Department of Endoscopy and Digestive System, Guizhou Provincial People’s Hospital, Guiyang, Guizhou, China; 3 Department of Respiratory and Critical Care Medicine, The First People’s Hospital of Yunnan Province, Kunming, Yunnan, China

**Keywords:** acetylshikonin, hepatic steatosis, metabolic dysfunction-associated steatotic liver disease, network pharmacology, peroxisome proliferator-activated receptor γ, traditional Chinese medicine

## Abstract

**Introduction:**

The liver, as the central metabolic hub of the body, is highly susceptible to diet-induced injury. The increasing prevalence of metabolic dysfunction-associated steatotic liver disease (MASLD) highlights the urgent need for effective clinical interventions. Currently, there are no specific therapeutics for MASLD, and dietary patterns are closely associated with its pathogenesis, making the exploration of natural bioactive compounds a promising strategy.

**Methods:**

In this study, we identified acetylshikonin (AS), a component derived from traditional Chinese medicine (TCM), as a core bioactive agent targeting MASLD via a cross-screening strategy of MASLD-related TCM formulas. Male mouse models of MASLD were induced by a high-fat and high-cholesterol (HFHC) diet or carbon tetrachloride (CCl4) and treated with AS (600 mg/kg, gavage) for six consecutive weeks. In vitro experiments were conducted on Hepa1-6 and HCCLM3 hepatocytes stimulated with palmitic acid/oleic acid (PA/OA, 1:2). Integrated network pharmacology, molecular docking, and thermal shift assays were applied to explore the underlying mechanism.

**Results:**

In vivo results showed that AS markedly attenuated hepatic steatosis (assessed by triglyceride and total cholesterol levels) and liver fibrosis (evaluated by collagen deposition). In vitro, AS suppressed intracellular lipid accumulation (validated by Oil Red O staining and lipid quantification) and inflammatory responses (assessed by pro-inflammatory cytokine expression) in the stimulated hepatocytes. Mechanistically, AS downregulated the transcriptional expression of key genes involved in lipid metabolism (Pparγ and Srebp1c), inflammation (Tnfα and Ccl2), and fibrosis (Col1a1 and Acta2) pathways. Integrated analyses confirmed peroxisome proliferator-activated receptor γ (PPARγ) as the core direct target of AS. Western blotting demonstrated that AS reduced PPARγ protein expression, and its lipid-lowering effect was synergistically enhanced when combined with the PPARγ antagonist GW9662.

**Discussion:**

This is the first study to definitively confirm that AS exerts therapeutic effects on diet-induced MASLD by targeting the PPARγ signaling pathway, thereby reducing hepatic lipid deposition, alleviating inflammation, and ameliorating liver fibrosis progression. Our findings provide novel experimental evidence supporting the use of natural products in MASLD treatment and lay a theoretical foundation for the application of AS in the health management of diet-related liver diseases.

## 1 Introduction

Metabolic dysfunction-associated steatotic liver disease (MASLD), previously known as non-alcoholic fatty liver disease (NAFLD), is the most common chronic liver disease worldwide, affecting approximately 25%–30% of adults. Its advanced subtype, metabolic dysfunction-associated steatohepatitis (MASH), can progress to end-stage liver diseases such as cirrhosis and hepatocellular carcinoma, threatening human health (Huang et al., 2025). MASLD is closely associated with obesity, insulin resistance, and metabolic syndrome and has long lacked approved drugs, with lifestyle interventions as the mainstay [European Association for the Study of the Liver (EASL and EASO, 2024)]. In March 2024, the FDA approved resmetirom (trade name: Rezdiffra), a liver-targeted THR-β agonist, for the treatment of non-cirrhotic MASH with moderate to advanced fibrosis (F2–F3 stages). As the first globally approved drug for MASH (Harrison et al., 2023), it confirms the feasibility of targeting liver metabolic pathways for MASH therapy (Chalasani et al., 2018). However, the pathophysiological mechanism of MASLD/MASH is complex, with limited response rates to single-drug therapy, and resmetirom requires more efficacy and safety data in broader populations (Wong et al., 2025). Thus, the development of new drugs targeting core pathogenic pathways such as lipid metabolism and inflammation remains urgently needed and clinically significant.

Natural products are an important source of drug discovery, with 60% of approved drugs derived directly or indirectly from them, featuring structural diversity, broad activities, and favorable safety profiles (Newman and Cragg, 2020). They hold great potential for MASLD with unmet therapeutic needs (Avery et al., 2014). Many natural compounds exert hepatoprotective effects by regulating key pathways. Peroxisome proliferator-activated receptor γ (PPARγ), a core regulator of lipid metabolism and inflammation, is a critical target for natural product development. For example, barley leaf metabolites can activate the PPARγ pathway to improve intestinal mucosal barrier function and alleviate inflammation (Yan et al., 2020), supporting that natural products exert anti-inflammatory and metabolic regulatory effects by targeting PPARγ. Others include puerarin-targeting GABRA1 to regulate the PPARγ-related gut–brain axis (Li et al., 2024) and berberine-regulating AKR1B10 to improve PPARγ-mediated metabolic disorders in NAFLD (Yang et al., 2024). In addition, mechanisms such as nuciferine–TFEB, rosmarinic acid–PRDX1, XLIX–WDR6, and HK–DDX5 (Shi et al., 2025; Yang et al., 2024; Yao et al., 2023; Zhang et al., 2023b) also provide new strategies for MASH treatment. Despite considerable progress, the mechanisms of action of most traditional Chinese medicine (TCM) formulas and natural compounds remain unclear.

Acetylshikonin (AS) is a naphthoquinone compound derived from *Lithospermum erythrorhizon*, with definite anti-inflammatory, antioxidant, and metabolic regulatory activities (Zeng et al., 2018). Natural products with such activities often regulate nuclear receptor pathways (including PPARγ) (Pan et al., 2022), providing a reasonable basis for AS to target PPARγ in MASLD treatment. Previous studies have shown that AS inhibits inflammation in COVID-19 mouse models by targeting the PLpro of SARS-CoV-2 (Lu et al., 2022) and exerts anti-proliferative effects in non-small-cell lung cancer by inhibiting the STAT3/EGFR pathway (Tang et al., 2022). Studies have confirmed that STAT3 and PPARγ have cross-regulation in inflammation and metabolic regulation (Serrano-Marco et al., 2012), suggesting that AS may be involved in PPARγ-mediated pathophysiological processes. In addition, AS can directly regulate hepatocyte function [e.g., inhibiting HepG2 cell proliferation and downregulating hepatic CYP2J2 activity (Park et al., 2017)]. In summary, the broad pharmacological potential of AS and its direct effect on liver cells support its research value in targeting PPARγ for anti-MASLD therapy. However, whether AS improves the core pathological features of MASLD (lipid accumulation, inflammation, and fibrosis) by regulating PPARγ and the underlying mechanisms remain unclear and urgently require in-depth investigation.

We integrated TCM formula screening, multi-omics analysis, and preclinical validation to explore the anti-MASLD potential of AS. Using palmitic acid-induced hepatocytes and HFHC/CCl_4_-induced MASLD mouse models, it was confirmed that AS alleviates hepatic steatosis through PPARγ-mediated transcriptional reprogramming of lipid metabolism. In this study, we combine traditional herbal medicine with modern molecular pharmacology, revealing that AS, as a dual-acting PPARγ modulator, can simultaneously improve metabolic abnormalities and fibrosis in MASLD, providing important evidence for the clinical translation of therapeutic strategies for this refractory disease.

## 2 Materials and methods

### 2.1 Chemicals and reagents

AS was purchased from MCE (Shanghai, China), and comfrey powder was purchased from Fsinuote (Baoji, Shanxi, China). For the animal experiments, comfrey powder was dissolved in saline and used to generate an AS diet. For cellular experiments, AS was dissolved in DMSO, prepared as a 1 mM storage solution, and stored in aliquots at −80 °C. The PPARG antagonist GW9962 was purchased from MCE (Shanghai, China).

### 2.2 Screening of active components and targets of TCM contained in four compounds

According to relevant TCM guidelines and professional websites, this study selected four recommended formulas for the treatment of non-alcoholic fatty liver disease: Zaozhu Yinchen decoction (ZZYCF), Qinggan Huatan Huoxue decoction (QGHTHXF), Yuganlong granules (YGLKL), and Ganning tablets (GNP). To identify their active ingredients, potential components with oral bioavailability (OB) ≥30% and drug-likeness (DL) ≥0.18 were first screened using the TCMSP (https://old.tcmsp-e.com/tcmsp.php, accessed on 5 June 2024) and SymMap (http://www.symmap.org/, accessed on 10 June 2024) databases. Subsequently, a Venn diagram was used to analyze the common active ingredients among the four formulas, ultimately identifying seven shared components, including AS. Furthermore, target prediction for these common ingredients was performed using the SwissTargetPrediction database (http://swisstargetprediction.ch/, accessed on 17 June 2024). The resulting protein targets were submitted to the UniProt database (http://www.uniprot.org, accessed on 25 June 2024) and standardized by applying a “*Homo sapiens*” filter. Finally, Cytoscape 3.7.2 was used to construct a target gene interaction network, systematically revealing the potential mechanisms of action.

### 2.3 Disease target prediction

Target information related to MASLD was collected from three databases: the DisGeNET database (https://www.disgenet.org/, accessed on 19 June 2024), the GeneCards database (http://www.genecards.org/, accessed on 19 June 2024) and the CTD database (https://ctdbase.org/, accessed on 19 June 2024). Relevant targets were retrieved using the search term “nonalcoholic fatty liver disease.” The disease targets in the CTD database were screened with an inference score greater than 20, and the disease targets in the GeneCards database were screened with a relevance score greater than the median.

The UniProt database was used to unify disease targets into gene symbols. Targets that are common in each database are screened out as therapeutic MASLD targets. Subsequently, the targets of MASLD were intersected with the targets of seven key active components, and the overlapping sites were selected as potential targets for MASLD intervention.

### 2.4 Construction of the PPI network

The shared drug–disease targets were imported into the STRING database (https://cn.string-db.org/, accessed on 27 June 2024) to construct a PPI network. Results were visualized using Cytoscape 3.7.2, and the network topology parameters (degree, betweenness, and closeness) for these targets were obtained using the NetworkAnalyst tool in Cytoscape.

### 2.5 Enrichment analysis

Core targets were subjected to Gene Ontology (GO) and Kyoto Encyclopedia of Genes and Genomes (KEGG) pathway enrichment analyses using the DAVID online platform (https://david.ncifcrf.gov/, accessed on 7 July 2024). The species was set to *H. sapiens*, and a significance threshold of *p* < 0.05 was applied. The results were sorted in a descending order based on *p*-values, and the top 10 entries for GO analysis and the top 20 entries for KEGG analysis were selected for visualization. We aimed to explore the potential roles and mechanisms of the related genes in biological processes, molecular functions, cellular components, and signaling pathways, providing an intuitive representation of the potential mechanisms by which seven shared active ingredients, including AS may treat MASLD.

### 2.6 Experimental animals and treatment

Eight-week-old male C57BL/6J mice were purchased from SPF (Beijing) Biotechnology Co., Ltd. and allowed to acclimate for 1 week.

The MASLD model was constructed using a high-fat and high-cholesterol (HFHC) diet. After 1 week of adaptive feeding, C57BL/6 mice were randomly divided into three groups (n = 6). The grouping and treatment regimens were as follows: (1) NCD group: normal chow diet + intragastric administration of normal saline for 6 weeks; (2) HFHC group: high-fat and high-cholesterol diet (fat content: 20% and cholesterol content: 2%) + intragastric administration of normal saline for 6 weeks; and (3) HFHC + AS group: high-fat and high-cholesterol diet + intragastric administration of AS (600 mg/kg/day) for 6 weeks.

A liver fibrosis model was established by intraperitoneal injection of carbon tetrachloride (CCl_4_). After 1 week of adaptive feeding, C57BL/6 mice were randomly divided into three groups (n = 6 per group), and all mice were fed a normal diet throughout the experiment. The grouping and treatment protocols were as follows: 1) normal chow diet (NCD) group: intraperitoneal injection of corn oil (twice a week) + intragastric administration of normal saline for 4 consecutive weeks; 2) carbon tetrachloride (CCl_4_) group: intraperitoneal injection of CCl_4_ (0.5 mL/kg per injection, twice a week) + intragastric administration of normal saline for 4 consecutive weeks; and 3) carbon tetrachloride + AS (CCl_4_ + AS) group: intraperitoneal injection of CCl_4_ (0.5 mL/kg per injection, twice a week) + intragastric administration of AS (600 mg/kg per day) for 4 consecutive weeks. For the convenience of injection, the CCl_4_ solution for intraperitoneal injection was prepared at a volume ratio of CCl_4_: corn oil = 1:9.

At the end of the treatment period, all mice were sacrificed. Liver tissues were harvested for morphological observation of hepatic injury and subsequent experimental assays. All mice were housed in an SPF-grade animal facility at the School of Medicine, Guizhou University, under standard conditions (25 °C, 12-h light/dark cycle, with free access to water). All experimental procedures involving animals were approved by the Ethics Committee for Animal Use and Care of Guizhou University and complied with relevant ethical guidelines (HMEE-GUZ-2024-T025). Liver tissues from ob/ob and db/db mice were generously provided by the laboratory of Professor Bin Liang at the Center for Life Sciences, Yunnan University.

### 2.7 Hematoxylin–eosin staining

The harvested liver tissue was fixed in 10% neutral buffered formalin, embedded in paraffin, and cut into 4-μm sections. Sections were stained with hematoxylin and eosin (H&E) using a commercial staining kit (SolarBio, G1120), according to the manufacturer’s instructions for histological assessment.

### 2.8 Oil Red O staining

Frozen sections were prepared from collected liver tissue and stained with Oil Red O using a commercial kit (G1262, SolarBio, China), according to the manufacturer’s instructions. The cellular Oil Red O staining procedure was also carried out according to the instructions.

### 2.9 Cell culture and treatment

Cells were cultured in DMEM (E600033, Sangon Biotech Co., Ltd., Shanghai, China) supplemented with 10% fetal bovine serum (164210-50, Procell Life Science and Technology Co., Ltd., Wuhan, China) and 1% penicillin–streptomycin (S110JV, Yuanpei Biotech Co., Ltd., Shanghai, China) and routinely incubated in a humidified incubator at 37 °C with 5% CO_2_; Hepa1-6 cells, HCCLM3 cells, and LX2 cells were kindly provided by the research group of Professor Bin Liang from the Center for Life Sciences, Yunnan University, China. The MASLD cell model was established by inducing Hepa1-6 cells or HCCLM3 cells with a mixture of palmitic acid (PA, H8780, SolarBio Science and Technology Co., Ltd., Beijing, China) and oleic acid (OA, 0108485, Aladdin Biochemical Technology Co., Ltd., Shanghai, China) at a final concentration of 0.6 mM (OA: PA = 2:1); the hepatocyte fibrosis model was constructed by incubating LX2 cells with 10 ng/mL transforming growth factor-β1 (TGF-β_1_) for 24 h; during modeling, different concentrations of AS were added to the administration groups for co-incubation.

### 2.10 CCK-8 assay

Hepa1-6 and HCCLM3 cells were seeded in 96-well plates at a density of 2.5 × 10^3^ cells per well and allowed to adhere overnight. The following day, the cells were treated with varying concentrations of AS (5, 10, 20, 30, 40, 50, 60, and 70 μM) for 24 h. A control group, treated with 0.1% DMSO (vehicle), was included to correspond to the 0 μM AS concentration. The CCK-8 Assay Kit (K1018, APExBIO, United States) was used to assess cell viability, according to the manufacturer’s instructions.

### 2.11 TG/TC assay

The contents of total cholesterol (TC) and triglyceride (TG) in hepatocytes were quantified using commercial detection kits (TC kit: Nanjing Jiancheng Bioengineering Institute, China, Cat. No. A111-1-1; TG kit: Nanjing Jiancheng Bioengineering Institute, China, Cat. No. A110-1-1), following the manufacturer’s standard protocols. In accordance with the kit instructions, the detection wavelengths for TC and TG were set to 450 nm and 510 nm, respectively.

### 2.12 Real-time quantitative PCR

Total RNA was extracted using RNAiso Plus (108-95-2, TaKaRa, Japan), and RNA quality was assessed using a Thermo NanoDrop 2000 Spectrophotometer. cDNA synthesis was performed using the PrimeScript RT Reagent Kit (RR036A, TaKaRa, Japan). Glyceraldehyde-3-phosphate dehydrogenase (GAPDH) was used as the internal control to normalize target gene expression. Relative expression levels were calculated using the 2^−ΔΔCt method. All analyses were performed independently in triplicate. Primer sequences are listed in Supplementary Table S1.

### 2.13 BODIPY staining

First, hepa1-6 or HCCLM3 cells were seeded onto coverslips placed in 24-well plates and treated with palmitic acid/oleic acid (PA/OA) and AS or other drugs 24 h later. After treatment, the cells were fixed with 4% paraformaldehyde in PBS solution for 1 h at room temperature or overnight at 4 °C. The coverslips were then washed with PBS. The coverslips were then washed three times with PBS for 5 min each time. Cells were stained with 10 μM BODIPY (121207-31-6, MCE, United States) for 30 min, followed by three washes with PBS. Finally, the coverslips were sealed with a sealer containing DAPI.

### 2.14 Molecular docking

Molecular docking was performed to further validate the interactions between AS and key target genes. The 2D structure of AS was downloaded from the PubChem database (https://pubchem.ncbi.nlm.nih.gov/, accessed on 9 March 2025) and converted to PDBQT format using ObGUI software. The structures of the target proteins were retrieved from the Protein Data Bank (PDB) (https://www.rcsb.org/, accessed on 9 March 2025). After removing water molecules, adding polar hydrogen, and calculating Kollman charges, simulation docking of bioactive compounds and target proteins was performed using AutoDock Vina 1.5.6 software. Docking scores lower than −5.0 kcal/mol were considered indicative of strong binding interactions between the compound and its targets. The best docking poses were visualized using PyMOL software.

### 2.15 Cellular thermal shift assay

Hepa1-6 cells were cultured under standard conditions (37 °C and 5% CO_2_) until reaching approximately 80% confluence and were then randomly divided into two groups: the control group and the AS-treated group. For the AS-treated group, cells were incubated with 4 μM AS at 37 °C for 2 h; the control group was treated with an equal volume of vehicle (dimethyl sulfoxide, DMSO) without AS. After treatment, cells were harvested and resuspended in phosphate-buffered saline (PBS) containing 1% protease inhibitor cocktail to prevent protein degradation. Cell lysis was performed using three consecutive freeze–thaw cycles (1 min of freezing in liquid nitrogen followed by 1 min of thawing in a 37 °C water bath per cycle). The cell lysates were centrifuged at 12,000 × g and 4 °C for 15 min, and the supernatants were collected.

Each supernatant sample was aliquoted into seven equal portions (50 μL per portion) and subjected to different temperature treatments (37, 40, 43, 46, 49, 52, and 55 °C) in a thermal cycler for 3 min. Immediately after heating, the samples were placed on ice for 5 min to terminate thermal denaturation, followed by centrifugation at 12,000 × g and 4 °C for 15 min to remove heat-aggregated insoluble proteins. The resulting supernatants (containing thermostable proteins) were mixed with 2× sodium dodecyl sulfate (SDS) loading buffer at a 1:1 volume ratio and boiled at 95 °C for 10 min to denature the proteins. The relative expression level of PPARγ in each sample was detected by Western blot analysis as described in Section 2.16.

### 2.16 Western blot analysis

Proteins were extracted from liver tissues (ob/ob mice and db/db mice liver tissues were generously provided by Prof. Bin Liang’s laboratory, School of Life Sciences, Yunnan University, China) or HCCLM3 cells using a high-potency RIPA solution, and the manufacturer’s procedure was strictly followed. Protein concentration was then quantified using the BCA Protein Assay Kit. A protein sample of 30 μg was added to each well of a 12% SDS-PAGE gel for electrophoresis. After separation, proteins were efficiently transferred to PVDF membranes (Millipore Corp, United States). To block non-specific binding, the membranes were incubated in 5% skimmed milk powder for 2 h. The membranes were incubated overnight at 4 °C with primary antibodies against PPARγ protein (16643-1-AP, 1:1000, Proteintech) and GAPDH (60004-1-Ig, 1:10,000, Proteintech). After washing the membrane three times, the membrane was incubated with the HRP-coupled secondary antibody (1:10,000, Azyme) for 1 h. Images were acquired using an automated chemiluminescence imaging system and analyzed using ImageJ 1.8.0.

### 2.17 Statistical analysis

Statistical analysis and graphing were performed using GraphPad Prism 8.0.2 software. Data are presented as the mean ± standard deviation (SD). A one-way analysis of variance (ANOVA) was used for comparisons among multiple groups, while the Student’s t-test was applied for comparisons between two groups. For statistical significance, *p* < 0.05 was considered statistically significant.

## 3 Results

### 3.1 Cross-formula screening identifies natural bioactive molecules for MASLD

**FIGURE 1:**
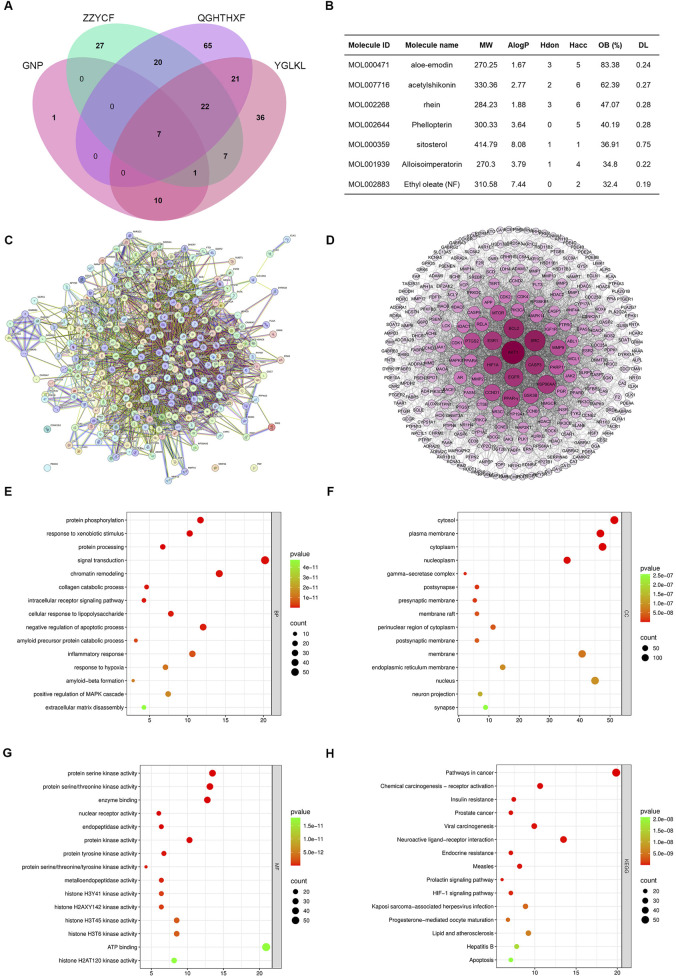
MASLD bioactive molecules identified via cross-formula screening. **(A,B)** Venn diagram illustrating seven shared compounds across Ganning tablets, Zaozhu Yinchen decoction, Qinggan Huatan Huoxue decoction, and Yuganlong granules. **(C)** Predicted targets for the seven compounds were subjected to protein–protein interaction (PPI) analysis using STRING. **(D)** Core targets identified using network centrality analysis. **(E)** GO biological process enrichment showing involvement in protein phosphorylation, inflammatory responses, and hypoxia responses. **(F)** GO molecular functions highlight protein kinase and ATP-binding activities. **(G)** Cellular component analysis indicating membrane and cytoplasm localization. **(H)** KEGG pathway enrichment showing associations with NF-κB signaling, insulin resistance, apoptosis, and lipid metabolism.

To discover natural products targeting MASLD with lipid-lowering effects, we analyzed classical TCM formulas from MASLD clinical guidelines. We identified small molecules consistently present across multiple formulae, specifically *Ganning* tablets, *Zaozhu Yinchen* decoction, *Qinggan Huatan Huoxue* decoction, and *Yuganlong granules*. Screening for DL and OB yielded 430 initial candidate compounds (Supplementary Table S2). Among these, seven constituents (aloe-emodin, AS, rhein, phellopterin, sitosterol, alloisoimperatorin, and ethyl oleate) were identified as common components shared across all four independent formulas (Figures 1A,B).

Furthermore, target prediction and analysis were performed for the seven compounds to elucidate their mechanisms of action. PPI network/hub gene analysis (Figures 1C,D) was followed by GO and KEGG enrichment analyses (Supplementary Table S3). GO analysis revealed significant associations with protein phosphorylation, inflammatory response, hypoxia response, and amyloid-beta processing, while molecular function enrichment highlighted protein kinase activities and ATP binding (Figures 1E–G). KEGG analysis identified key pathways, including pathways in cancer, chemical carcinogenesis, lipid and atherosclerosis, insulin resistance, NF-κB signaling, and apoptosis (Figure 1H). These findings indicate that the compounds likely ameliorate MASLD by modulating targets involved in NF-κB signaling, inflammation, apoptosis, and insulin resistance. Among the seven shared components, AS stood out due to its superior drug-likeness and oral bioavailability. Given its favorable pharmacokinetic properties and the unclear role of its mechanism in MASLD, we prioritized AS for in-depth mechanistic investigation.

### 3.2 AS alleviates HFHC diet-induced metabolic dysfunction

**FIGURE 2:**
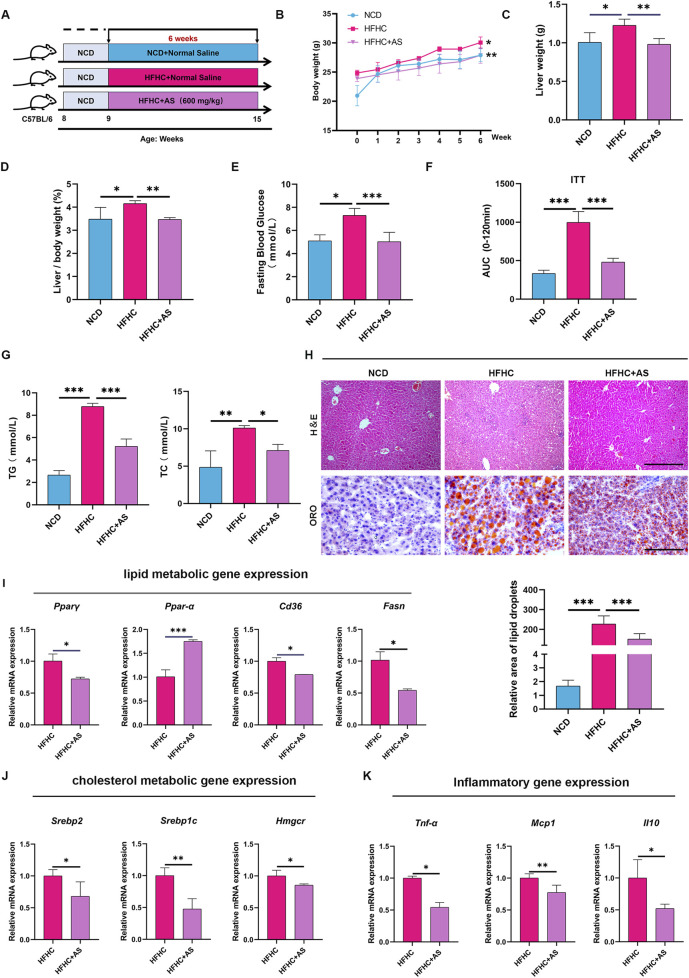
AS alleviates HFHC-induced metabolic dysfunction. **(A)** Schematic diagram of *in vivo* experimental design. **(B,C)** AS markedly attenuated the increases in body weight and liver weight in mice fed an HFHC diet. **(D)** Ratio of liver weight to body weight in mice. **(E)** Fasting blood glucose levels. **(F)** Assessment of insulin resistance. **(G)** Contents of triglycerides (TGs) and total cholesterol in liver tissues. **(H)** Representative images of H&E staining and Oil Red O staining, as well as quantification of the lipid area in liver tissues. Scale bar: 100 μm. **(I,J)** mRNA expression levels of lipid metabolism-related genes (*Pparγ*, *Pparα*, *Cd36*, *Srebp1c*, *Srebp2*, and *Hmgcr*) in liver tissues. **(K)** mRNA expression levels of inflammatory factors (*Tnfα*, *Mcp1*, and *Il10*) in the livers of AS-treated mice. Data are expressed as the mean ± SD; one-way ANOVA with Tukey’s *post hoc* test was used for statistical comparison. All experimental data are presented as the mean ± standard deviation (SD). n = 6. Statistical significance was defined as *p < 0.05, **p < 0.01, and ***p < 0.001.

To explore the potential of acetylshikonin (AS) in improving MASLD, we established a liver injury and metabolic disorder model in male C57BL/6J mice using an HFHC diet, followed by 6 weeks of AS intervention (Figure 2A). The results showed that compared with the HFHC control group, AS treatment markedly inhibited the increases in body weight and liver weight induced by an HFHC diet (Figures 2B,C) and improved HFHC-induced hepatic steatosis, as manifested by a decrease in the liver-to-body weight ratio (LW/BW) (Figure 2D). Meanwhile, AS treatment could reduce blood glucose levels, alleviate insulin resistance (Figures 2E,F), and decrease hepatic triglyceride and total cholesterol contents (Figure 2G). H&E and Oil Red O staining of liver tissues further confirmed that AS could significantly reduce intrahepatic lipid deposition (Figure 2H). At the molecular mechanism level, AS could significantly regulate the mRNA expression levels of key hepatic lipid metabolism regulators (*Pparγ*, *Pparα*, *Cd36*, *Srebp2*, *Srebp1c*, and *Hmgcr*) (Figures 2I,J), and significantly downregulate the mRNA expression of inflammation-related genes (*Tnfα*, *Mcp1*, and *Il10*) (Figure 2K).

Liver fibrosis is a critical progressive stage of fatty liver disease. To clarify the role of AS in liver fibrosis, we established a CCl_4_-induced *in vivo* liver fibrosis model (Supplementary Figure S1A). Sirius Red staining confirmed that AS could significantly improve CCl_4_-induced liver fibrosis (Supplementary Figure S1B). In *in vitro* experiments, LX-2 hepatic stellate cells (HSCs) were stimulated with TGF-β_1_ to establish an activation model. Quantitative real-time polymerase chain reaction (qPCR) results showed that AS could markedly inhibit the upregulation of mRNA expression of key activation markers of hepatic stellate cells induced by TGF-β_1_ (*Col1a1*, *Col3a1*, *Acta2*, and *Tgfb*_*1*_) (Supplementary Figure S2). In summary, AS can effectively improve HFHC diet-induced hepatic steatosis and alleviate CCl_4_-induced liver fibrosis.

### 3.3 AS suppresses lipid accumulation and inflammation in PA/OA-treated Hepa1-6 hepatocytes

**FIGURE 3:**
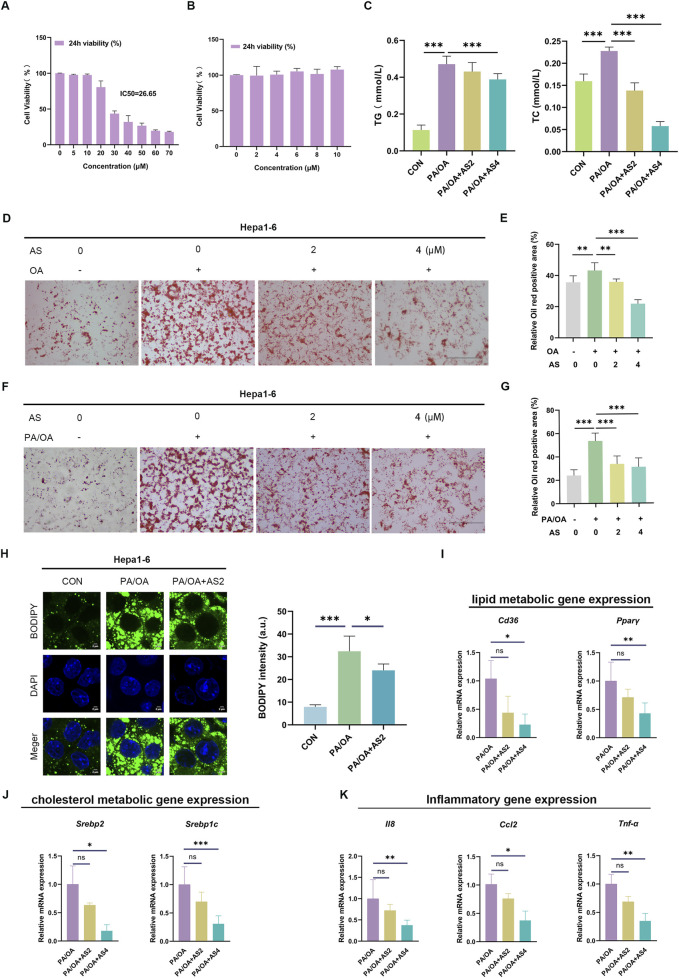
AS suppresses lipid accumulation and inflammation in Hepa1-6 cells **(A)** Cell viability of Hepa1-6 hepatocytes treated with increasing concentrations of AS for 24 h was assessed using a CCK-8 assay to determine the IC_50_ value. **(B)** Cell viability of Hepa1-6 cells co-treated with PA/OA and AS at concentrations of 0, 2, 4, 6, 8, and 10 μM over a 24-h period was assessed using the CCK-8 assay. **(C)** Intracellular triglyceride (TG) and total cholesterol (TC) levels were measured in PA/OA-induced Hepa1-6 cells with or without AS treatment (2–4 μM) using biochemical assays. **(D–G)** Lipid accumulation was evaluated using Oil Red O staining. Representative images; scale bar: 100 μm. **(H)** Lipid droplet number and size were visualized using BODIPY 493/503 staining; fluorescence intensity was quantified using ImageJ, showing a marked decrease in AS-treated cells; scale bar: 2 μm. **(I–K)** qPCR analysis of lipid metabolism genes (*Cd36* and *Pparγ*), cholesterol regulators (*Srebp1c* and *Srebp2)*, and pro-inflammatory cytokines (*Il-8*, *Ccl2*, and *Tnfα*) after AS treatment in PA/OA-induced Hepa1-6 cells. Data are presented as the mean ± SD. n = 3. *p < 0.05, **p < 0.01, and ***p < 0.001.

Building on these findings, we further explored the effects of AS on hepatic cells by delineating its impact on Hepa1-6 hepatocytes, focusing on cell viability, lipid accumulation, and transcriptional regulation. AS demonstrated dose-dependent cytotoxicity over 24 h (IC_50_ = 26.65 μM; Figure 3A). Meanwhile, the planned experimental concentrations of AS, when combined with PA/OA, did not exert cytotoxic effects on cells (Figure 3B). Treatment with AS (2–4 μM) markedly attenuated PA/OA-induced elevations in TG and TC levels (Figures 3B,C). Oil Red O staining corroborated these findings, revealing a marked reduction in the relative area of lipid-positive deposits following AS administration (Figures 3D–G). Complementary BODIPY staining visualized a substantial decrease in both the number and size of intracellular lipid droplets upon AS treatment, with quantitative fluorescence analysis confirming significantly lower intensity compared to PA/OA controls (Figure 3H). Furthermore, AS elicited coordinated downregulation of key pathological genes: lipid metabolism regulators *Cd36* and *Pparγ* (Figure 3I); cholesterol biosynthesis mediators *Srebp2* and *Srebp1c* (Figure 3J); and inflammatory cytokines *Il-8 Ccl2* and *Tnfα* (Figure 3K). Collectively, these data demonstrate that AS suppresses lipid accumulation and inflammation in hepatocytes, demonstrating therapeutic potential for MASLD.

### 3.4 AS reduces lipid accumulation and droplet formation in human-derived HCCLM3 cells

**FIGURE 4:**
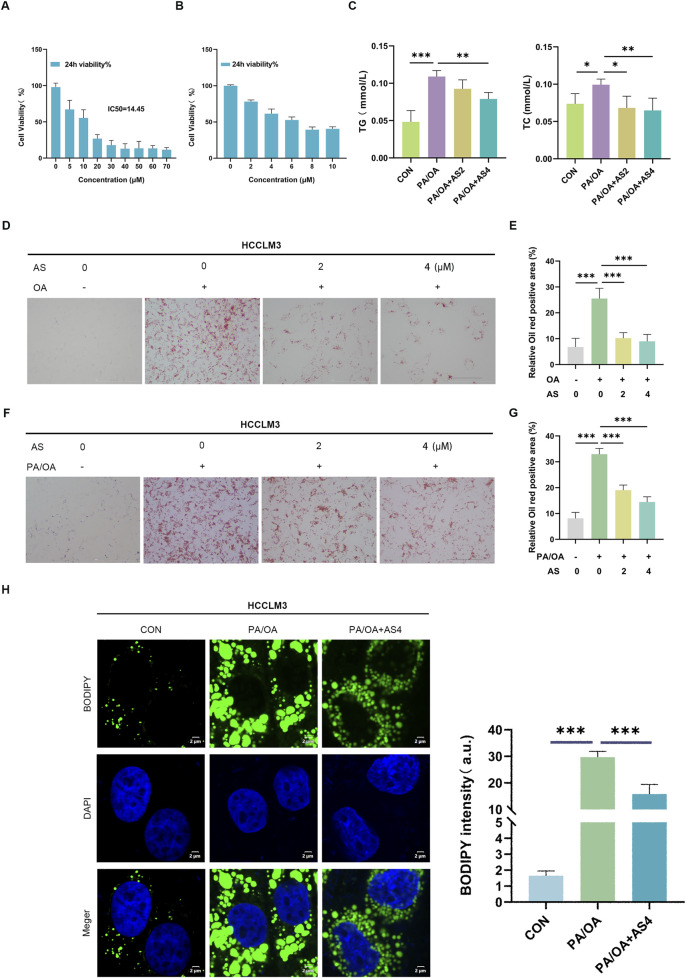
AS reduces lipid accumulation/droplet formation in HCCLM3 cells **(A)** Cytotoxicity of AS in HCCLM3 cells after 24 h was determined using the CCK-8 assay. **(B)** Cell viability of HCCLM3 cells co-treated with PA/OA and AS at concentrations of 0, 2, 4, 6, 8, and 10 μM over a 24-h period was assessed using the CCK-8 assay. **(C)** Quantification of intracellular TG and TC levels in PA/OA-treated HCCLM3 cells with or without AS (2–4 μM) intervention using enzymatic assays. **(D–G)** Representative images and quantification of Oil Red O staining demonstrating AS-mediated reduction in lipid accumulation; scale bar: 100 μm. **(H)** BODIPY staining and corresponding fluorescence intensity analysis confirmed decreased lipid droplet content in AS-treated HCCLM3 cells; scale bar: 2 μm. Data are presented as the mean ± SD. n = 3. *p < 0.05, **p < 0.01, and ***p < 0.001.

To comprehensively evaluate the lipid-lowering potential of AS for MASLD, we extended our investigation to human cellular models. In particular, AS exhibited dose-dependent cytotoxic effects in human HCCLM3 cells, with a 24-h IC_50_ value of 14.45 μM (Figure 4A). Meanwhile, the planned experimental concentrations of AS, when combined with PA/OA, did not exert cytotoxic effects on cells (Figure 4B). Following PA/OA induction, AS treatment (2–4 μM) markedly attenuated intracellular TG and TC contents (Figure 4C). Oil Red O staining confirmed markedly reduced lipid deposition, with AS treatment decreasing the relative stained area by 58.3% at 4 μM (Figures 4D–G). Complementary fluorescence imaging demonstrated that AS substantially reduced both the number and size of lipid droplets, as visualized using BODIPY staining. Quantitative analysis revealed lower BODIPY fluorescence intensity in AS-treated cells versus PA/OA controls (Figure 4H). Collectively, these results demonstrate that AS effectively reduces lipid accumulation in PA/OA-treated human HCCLM3 cells, further supporting its potential role in MASLD.

### 3.5 Integrative network analysis reveals key targets underlying AS therapeutic effects in MASLD

**FIGURE 5:**
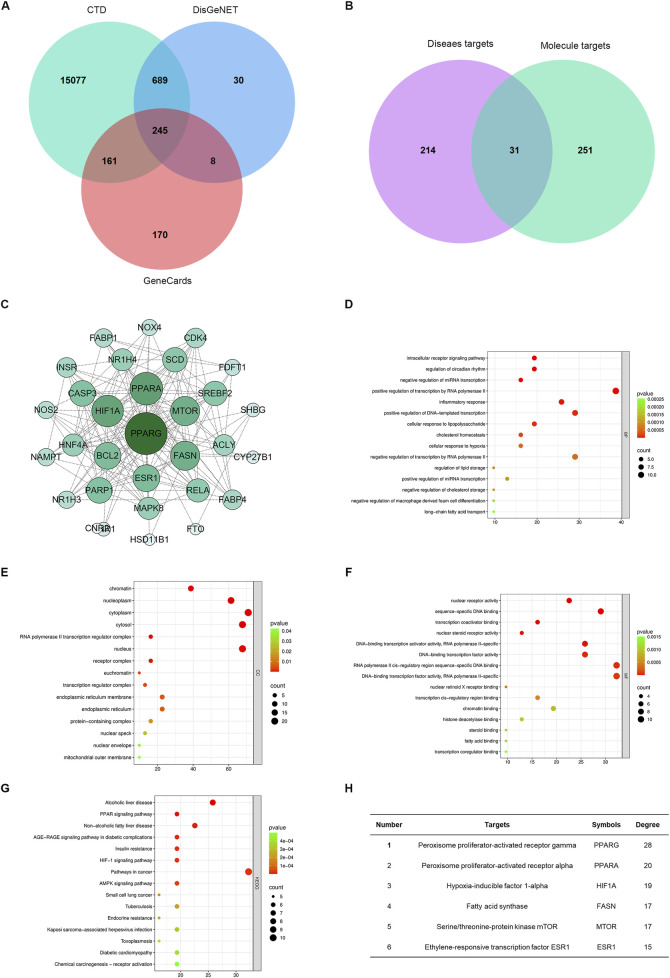
Network analysis reveals AS therapeutic targets in MASLD. **(A)** Venn diagram showing the number of overlapping genes among MASLD-related targets from CTD, DisGeNET, and GeneCards. **(B)** Identification of 31 overlapping genes between AS- and MASLD-related targets. **(C)** PPI network of shared genes constructed using STRING and Cytoscape, highlighting core nodes including *Pparγ*, *Pparα*, *Fasn*, and *Hif1a*. **(D–G)** GO and KEGG enrichment analyses of overlapping targets indicate major involvement in fatty acid metabolism, inflammation, and hypoxia pathways. **(H)** Top six core genes ranked by degree centrality in the PPI network.

Building on the above findings, we further investigated the potential mechanisms by which AS exerts its therapeutic effects in MASLD. To this end, we systematically integrated data from multiple databases (CTD, DisGeNET, and GeneCards) to analyze the overlap between MASLD-related genes and AS targets. As shown in Figure 5A, a total of 245 genes were commonly identified across all three databases, highlighting their potential significance in MASLD pathogenesis (Supplementary Table S4). Further analysis revealed that 245 genes were associated with MASLD, while 282 were related to AS, with 31 overlapping genes implicated in both disease progression and AS pharmacological activity (Figure 5B; Supplementary Table S5). To better understand the functional relevance of these intersecting genes, we constructed a core gene interaction network, identifying central nodes such as *Pparγ*, *Pparα*, *mTOR*, *Fasn*, *Hif1a*, *Esr1*, *and Bcl2*, which are key regulators of lipid metabolism, inflammation, and cell proliferation (Figure 5C). Functional enrichment analysis further demonstrated that these genes participate in critical biological processes and signaling pathways: for instance, *Pparγ* and *Fasn* are linked to fatty acid metabolism, *Hif1a* to hypoxia responses, and *Esr1* to nuclear receptor activity (Figures 5D–G; Supplementary Table S6). Additionally, Figure 5H highlights the top-ranked genes based on the network degree, underscoring the pivotal roles of *Pparγ*, *Pparα*, and *Hif1a*. Collectively, these integrative analyses provide mechanistic insights into the multi-target actions of AS in MASLD and support its therapeutic potential.

### 3.6 AS attenuates hepatic steatosis through the PPARγ pathway

**FIGURE 6:**
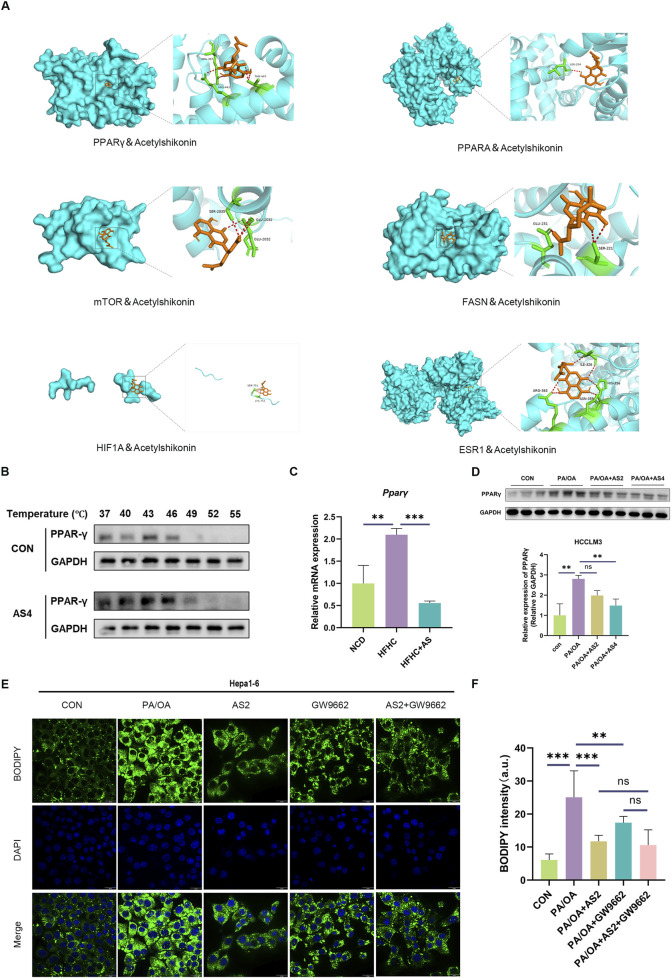
AS attenuates steatosis via the multi-target and PPARγ pathways. **(A)** Molecular docking models showing AS binding to key targets including PPARγ, PPARα, mTOR, FASN, HIF1A, and ESR1, generated using AutoDock Vina. **(B)** Thermal shift assay showing increased thermal stability of recombinant PPARγ protein upon AS binding at various temperatures. **(C)** qPCR analysis showing reduced hepatic Pparγ mRNA levels in AS-treated HFHC-fed mice (n = 6). **(D)** Western blot analysis demonstrating dose-dependent downregulation of the PPARγ protein in PA/OA-stimulated Hepa1-6 cells treated with AS (n = 3); representative blots and quantification are shown. **(E,F)** Lipid droplet accumulation assessed using BODIPY staining in Hepa1-6 cells co-treated with AS and the PPARγ antagonist GW9662; fluorescence quantification confirms the synergistic effect; scale bar: 10 μm. All quantitative data are shown as the mean ± SD; *p < 0.05, **p < 0.01, and ***p < 0.001.

To validate these network predictions, we performed molecular docking analysis between AS and core regulators (*Pparγ*, *Pparα*, *mTOR*, *Fasn*, *Hif1a*, and *Esr1*) identified in the gene interaction network. Figure 6A illustrates the binding modes of AS with these targets, demonstrating favorable binding affinity to all six targets. Molecular docking results demonstrated strong binding affinities between AS and all target proteins, validating the rationality of our screening strategy (Supplementary Figure S3). Based on the interaction networks identified in Figures 5C, 6A, we next directed our attention to the AS–PPAR signaling axis as a potential mechanistic pathway. In two widely used MASLD models, *ob/ob* and *db/db* mice, Western blot analysis revealed upregulation of PPARγ expression compared to wild-type controls (Supplementary Figure S4A,B), highlighting its pathological relevance.

To further elucidate the mechanism of action of AS, we investigated its modulatory effects on PPARγ. Figure 6C further revealed that AS significantly enhanced the thermal stability of PPARγ across various temperatures. To validate the regulatory effect of AS on PPARγ expression, we first assessed *Pparγ* mRNA levels in the livers of HFHC-fed mice treated with AS. The results showed that AS significantly downregulated *Pparγ* expression compared to the HFHC group (Figure 5C). Furthermore, in cellular models, Western blot analysis demonstrated that AS dose-dependently reduced PPARγ protein expression (Figure 5D). Consistently, co-administration of the PPARγ antagonist GW9662 potentiated the lipid-lowering efficacy of AS, manifested as markedly reduced lipid droplet accumulation *in vitro* (Figures 6E,F). These results definitively establish the involvement of PPARγ in the mechanism of action of AS, indicating that its therapeutic efficacy against MASLD operates through suppression of the PPARγ pathway.

## 4 Discussion

MASLD has emerged as an increasingly severe global public health challenge. Its pathophysiological process involves complex interactions among multiple links, including lipid metabolism disorders, chronic inflammation, and liver fibrosis. Currently, clinical treatment options are scarce, with only resmetirom approved for the treatment of MASH with F2–F3 fibrosis, which makes it difficult to meet the full-course treatment needs (Harrison et al., 2023; Zhang et al., 2025). Therefore, the development of novel therapeutic drugs that can simultaneously target multiple pathological links of MASLD is of great clinical significance. TCM exhibits tremendous potential in the treatment of complex metabolic diseases due to its unique advantages, including multi-component and multi-target regulation. However, unclear efficacy material basis and ambiguous mechanisms of action severely hinder its clinical translation process (Shi et al., 2012; Xue et al., 2025). AS, screened in this study using a “cross-formula common component mining” strategy, showed considerable therapeutic effects in improving MASLD in both *in vivo* and *in vitro* experiments, providing new clues for TCM-inspired drug development for MASLD.

We adopted an HFHC diet-induced MASLD mouse model and a CCl_4_-induced liver fibrosis mouse model to comprehensively evaluate the therapeutic potential of AS. The HFHC model simulates the most common diet-related pathogenic scenario of human MASLD and can reproduce core metabolic abnormalities such as obesity, insulin resistance, and hepatic lipid accumulation (Bhattacharjee et al., 2017). In contrast, the CCl_4_ model focuses on liver fibrosis, a key progressive stage of MASLD, which simulates the disease progression process by inducing oxidative stress and activation of HSCs (Ravichandra and Schwabe, 2021). The results showed that AS not only effectively improved HFHC diet-induced metabolic disorders, reduced hepatic lipid deposition, and improved insulin resistance but also markedly alleviated CCl_4_-induced liver fibrosis and inhibited the expression of HSC activation markers. This suggests that AS has regulatory effects on the metabolic initiation stage and progressive complications of MASLD, a characteristic that distinguishes it from single-target drugs and makes it more consistent with the complex pathophysiological characteristics of MASLD. In *in vitro* experiments, AS could dose-dependently reduce lipid deposition in PA/OA-stimulated hepatocytes at non-cytotoxic concentrations, further verifying the directness of its lipid-lowering effect. However, it should be noted that the human HCCLM3 cell line used in this study is a hepatocellular carcinoma cell line. Although it retains hepatocyte-like metabolic characteristics, its signaling pathways may be abnormal. Moreover, the monoculture model cannot reproduce the complex communication between parenchymal and non-parenchymal cells in the liver microenvironment. These limitations may affect the translational value of the results (Zheng et al., 2022). Future studies should use primary human hepatocytes and 3D liver organoid models for in-depth verification to more accurately evaluate the clinical application potential of AS.

At the mechanistic level, this study is the first to confirm that PPARγ is the core target of AS in exerting anti-MASLD effects. As a ligand-activated nuclear receptor, PPARγ plays a contradictory role in the pathophysiology of MASLD: on the one hand, it regulates adipocyte differentiation and systemic insulin sensitivity (Tontonoz et al., 1994); on the other hand, overexpression of PPARγ in the liver promotes *de novo* lipogenesis (DNL) and triglyceride accumulation, exacerbating hepatic steatosis (Zhang et al., 2023a). Previous studies have shown that systemic PPARγ agonists (such as thiazolidinediones) can improve insulin resistance but may aggravate hepatic steatosis, limiting their clinical application (He et al., 2016). In this study, multiple technical methods, including network pharmacology, molecular docking, and cellular thermal shift assay (CETSA), clarified the direct physical interaction between AS and PPARγ. Functional experiments further confirmed that AS can downregulate the expression of PPARγ in the liver and hepatocytes, inhibiting its transcriptional activity, and the combined use with the PPARγ antagonist GW9662 can synergistically enhance the lipid-lowering effect, suggesting that PPARγ inhibition is the key mechanism of AS’s action. It is worth noting that AS can not only downregulate the expression of DNL-related genes (*Srebp1c* and *Fasn*) by inhibiting the PPARγ/SREBP-1c pathway to reduce lipid synthesis and accumulation but also inhibit the expression of PPARγ-regulated inflammation-related genes (*Tnfα*, *Ccl2*, and *Il-8*) and (Figures 2I,J) fibrosis-related genes (*Col1a1* and *Acta2*), achieving synchronous regulation of multiple pathological links of MASLD (Supplementary Figure S2). More importantly, AS prevents the adverse effects of systemic PPARγ agonists and may exert its effect by selectively targeting hepatic PPARγ, a unique advantage that lays the foundation for its clinical application.

In addition, the “cross-formula common component mining” strategy adopted in this study also provides a useful reference for the modernization research of TCM. By screening common active components in clinically effective formulas, this strategy can quickly lock core compounds with potential therapeutic effects, effectively improve the efficiency and pertinence of TCM active component screening, and bridge the gap between the empirical application of TCM and modern molecular pharmacology. This study still has certain limitations: in addition to the deficiencies of the aforementioned cell models, the *in vivo* pharmacokinetic characteristics of AS need further optimization, its efficacy in samples from patients with different stages of MASLD has not been verified, and the combined application effect with existing therapeutic drugs also needs to be explored. Future research can focus on the structural modification of AS to improve its bioavailability and targeting, carry out *in vitro* experiments based on clinical samples and prospective clinical studies, and explore the combined treatment scheme of AS with existing drugs such as resmetirom to further enhance its therapeutic effect and provide new strategies and options for the clinical treatment of MASLD.

## 5 Conclusion

**FIGURE 7:**
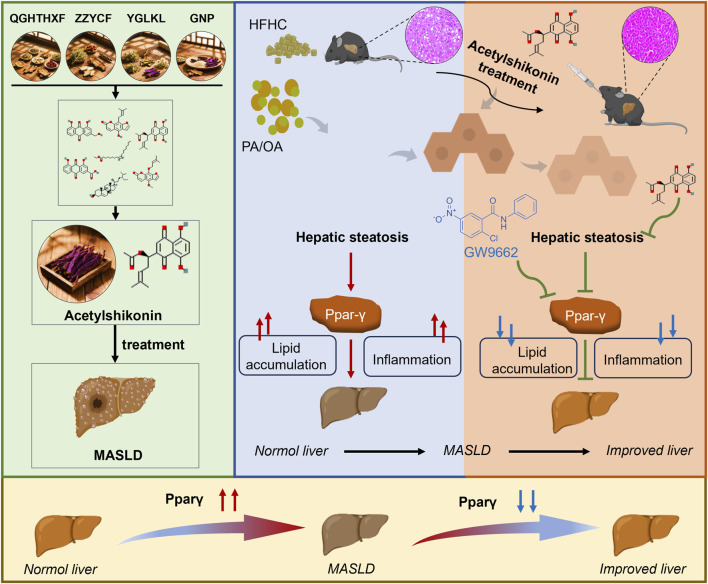
Proposed mechanism of AS in ameliorating MASLD. Cross-formula screening identifies AS as a shared bioactive constituent in MASLD-relevant TCM formulae. Experimental validation demonstrates that AS alleviates hepatic steatosis and inflammation *in vivo* and *in vitro*, with PPARγ serving as the central target mediating these therapeutic effects.

In summary, our findings support the proposed mechanism (Figure 7), in which cross-formula screening identifies AS as a shared bioactive constituent in MASLD-relevant TCM formulas. *In vivo* and *in vitro* findings demonstrate that AS ameliorates hepatic steatosis, inflammation, and fibrosis. Network pharmacology and experimental validations revealed that PPARγ serves as a central target mediating the beneficial effects of AS. These results establish AS as a promising therapeutic candidate and validate cross-formula screening as an effective strategy for ethnopharmacological discovery.

## Data Availability

The raw data supporting the conclusions of this article will be made available by the authors, without undue reservation.

## References

[B1] Avery V. M. Bashyam S. Burrows J. N. Duffy S. Papadatos G. Puthukkuti S. (2014). Screening and hit evaluation of a chemical library against blood-stage Plasmodium falciparum. Malar. J. 13, 190. 10.1186/1475-2875-13-190 24886460 PMC4094919

[B2] Bhattacharjee J. Kirby M. Softic S. Miles L. Salazar-Gonzalez R. M. Shivakumar P. (2017). Hepatic natural killer T-cell and CD8+ T-cell signatures in mice with nonalcoholic steatohepatitis. Hepatol. Commun. 1 (4), 299–310. 10.1002/hep4.1041 29152605 PMC5687094

[B3] Chalasani N. Younossi Z. Lavine J. E. Charlton M. Cusi K. Rinella M. (2018). The diagnosis and management of nonalcoholic fatty liver disease: practice guidance from the American Association for the Study of Liver Diseases. Hepatology 67 (1), 328–357. 10.1002/hep.29367 28714183

[B4] Easl E. , EASO European Association for the Study of Obesity EASO (2024). EASL-EASD-EASO Clinical Practice Guidelines on the management of metabolic dysfunction-associated steatotic liver disease (MASLD). J. Hepatol. 81 (3), 492–542. 10.1016/j.jhep.2024.04.031 38851997

[B5] Harrison S. A. Taub R. Neff G. W. Lucas K. J. Labriola D. Moussa S. E. (2023). Resmetirom for nonalcoholic fatty liver disease: a randomized, double-blind, placebo-controlled phase 3 trial. Nat. Med. 29 (11), 2919–2928. 10.1038/s41591-023-02603-1 37845512 PMC10667098

[B6] He L. Liu X. Wang L. Yang Z. (2016). Thiazolidinediones for nonalcoholic steatohepatitis: a meta-analysis of randomized clinical trials. Med. Baltim. 95 (42), e4947. 10.1097/md.0000000000004947 27759627 PMC5079311

[B7] Huang D. Q. Wong V. W. S. Rinella M. E. Boursier J. Lazarus J. V. Yki-Järvinen H. (2025). Metabolic dysfunction-associated steatotic liver disease in adults. Nat. Rev. Dis. Prim. 11 (1), 14. 10.1038/s41572-025-00599-1 40050362

[B8] Li Z. Cao W. Zhang Y. Lai S. Ye Y. Bao J. (2024). Puerarin ameliorates non-alcoholic fatty liver disease by inhibiting lipid metabolism through FMO5. Front. Pharmacol. 15, 1423634. 10.3389/fphar.2024.1423634 39055493 PMC11269101

[B9] Lu N. Gu T. Tian X. Zhao S. Jin G. Mangaladoss F. (2022). Acetylshikonin inhibits inflammatory responses and papain-like protease activity in murine model of COVID-19. Signal Transduct. Target Ther. 7 (1), 371. 10.1038/s41392-022-01220-7 36302747 PMC9610350

[B10] Newman D. J. Cragg G. M. (2020). Natural products as sources of new drugs over the nearly four decades from 01/1981 to 09/2019. J. Nat. Prod. 83 (3), 770–803. 10.1021/acs.jnatprod.9b01285 32162523

[B11] Pan J. Zhou W. Xu R. Xing L. Ji G. Dang Y. (2022). Natural PPARs agonists for the treatment of nonalcoholic fatty liver disease. Biomed. and Pharmacother. 151, 113127. 10.1016/j.biopha.2022.113127 35598367

[B12] Park S. H. Phuc N. M. Lee J. Wu Z. Kim J. Kim H. (2017). Identification of acetylshikonin as the novel CYP2J2 inhibitor with anti-cancer activity in HepG2 cells. Phytomedicine 24, 134–140. 10.1016/j.phymed.2016.12.001 28160853

[B13] Ravichandra A. Schwabe R. F. (2021). Mouse models of liver fibrosis. Methods Mol. Biol. 2299, 339–356. 10.1007/978-1-0716-1382-5_23 34028753

[B14] Serrano-Marco L. Barroso E. El Kochairi I. Palomer X. Michalik L. Wahli W. (2012). The peroxisome proliferator-activated receptor (PPAR) β/δ agonist GW501516 inhibits IL-6-induced signal transducer and activator of transcription 3 (STAT3) activation and insulin resistance in human liver cells. Diabetologia 55 (3), 743–751. 10.1007/s00125-011-2401-4 22179221

[B15] Shi K.-Q. Fan Y.-C. Liu W.-Y. Li L.-F. Chen Y.-P. Zheng M.-H. (2012). Traditional Chinese medicines benefit to nonalcoholic fatty liver disease: a systematic review and meta-analysis. Mol. Biol. Rep. 39 (10), 9715–9722. 10.1007/s11033-012-1836-0 22718512

[B16] Shi X. Bai Z. Lin Z. Zhang M. Xie X. Wang X. (2025). Rosmarinic acid confers beneficial effects by specifically activating PRDX1 peroxidase activity. Biochem. Biophysical Res. Commun. 779, 152457. 10.1016/j.bbrc.2025.152457 40779977

[B17] Tang Y. Wang Y. Wang X. Zhao Z. Cai H. Xie M. (2022). Acetylshikonin exerts anti-tumor effects on non-small cell lung cancer through dual inhibition of STAT3 and EGFR. Phytomedicine 101, 154109. 10.1016/j.phymed.2022.154109 35526322

[B18] Tontonoz P. Hu E. Graves R. A. Budavari A. I. Spiegelman B. M. (1994). mPPAR gamma 2: tissue-specific regulator of an adipocyte enhancer. Genes Dev. 8 (10), 1224–1234. 10.1101/gad.8.10.1224 7926726

[B19] Wong S. W. Yang Y. Y. Chen H. Xie L. Shen X. Z. Zhang N. P. (2025). New advances in novel pharmacotherapeutic candidates for the treatment of metabolic dysfunction-associated steatohepatitis (MASH) between 2022 and 2024. Acta Pharmacol. Sin. 46 (5), 1145–1155. 10.1038/s41401-024-01466-7 39870846 PMC12032127

[B20] Xue J. Gao S. You Z. Xu S. Zhou J. Jiang H. (2025). *In vitro* technology and ADMET research in traditional Chinese medicine. Front. Pharmacol. 16, 1605330. 10.3389/fphar.2025.1605330 40703346 PMC12283783

[B21] Yan T. Yan N. Wang P. Xia Y. Hao H. Wang G. (2020). Herbal drug discovery for the treatment of nonalcoholic fatty liver disease. Acta Pharm. Sin. B 10 (1), 3–18. 10.1016/j.apsb.2019.11.017 31993304 PMC6977016

[B22] Yang S. Cao S. J. Li C. Y. Zhang Q. Zhang B. L. Qiu F. (2024). Berberine directly targets AKR1B10 protein to modulate lipid and glucose metabolism disorders in NAFLD. J. Ethnopharmacol. 332, 118354. 10.1016/j.jep.2024.118354 38762210

[B23] Yao Z. Gong Y. Chen W. Shao S. Song Y. Guo H. (2023). Upregulation of WDR6 drives hepatic *de novo* lipogenesis in insulin resistance in mice. Nat. Metab. 5 (10), 1706–1725. 10.1038/s42255-023-00896-7 37735236 PMC10590755

[B24] Zeng J. Zhu B. Su M. (2018). Autophagy is involved in acetylshikonin ameliorating non-alcoholic steatohepatitis through AMPK/mTOR pathway. Biochem. Biophysical Res. Commun. 503, 1645–1650. 10.1016/j.bbrc.2018.07.094 30055803

[B25] Zhang Y. Xu J. Zhou D. Ye T. Zhou P. Liu Z. (2023a). Swimming exercise ameliorates insulin resistance and nonalcoholic fatty liver by negatively regulating PPARγ transcriptional network in mice fed high fat diet. Mol. Med. 29 (1), 150. 10.1186/s10020-023-00740-4 37907845 PMC10617119

[B26] Zhang Y. Ye S. Lu W. Zhong J. Leng Y. Yang T. (2023b). RNA helicase DEAD-box protein 5 alleviates nonalcoholic steatohepatitis progression *via* tethering TSC complex and suppressing mTORC1 signaling. Hepatology 77 (5), 1670–1687. 10.1002/hep.32651 35796622

[B27] Zhang F. Meng Y. Zhao B. Li Z. Han M. Li M. (2025). Resmetirom: the first FDA-Approved drug for Metabolic dysfunction-associated steatohepatitis (MASH) with a perspective on precision medicine. Clin. Drug Investig. 45 (11), 837–845. 10.1007/s40261-025-01484-1 40986164

[B28] Zheng Y. B. Ma L. D. Wu J. L. Wang Y. M. Meng X. S. Hu P. (2022). Design and fabrication of an integrated 3D dynamic multicellular liver-on-a-chip and its application in hepatotoxicity screening. Talanta 241, 123262. 10.1016/j.talanta.2022.123262 35144112

