# Supplementary material for: Acetylshikonin mitigates diet-induced MASLD by targeting PPARγ-mediated metabolic dysfunction
Source: Front Pharmacol. 2026 Feb 23;17:1735481. doi: 10.3389/fphar.2026.1735481 (PMC12968015; doi:10.3389/fphar.2026.1735481)
Supplement: Supplementary file 3 [file Supplementaryfile1.docx]

**Supplementary Figures**

**Acetylshikonin a bioactive naphthoquinone from traditional Chinese medicine formulae improves MASLD via PPAR gamma modulation**

Ling Ou^a^*, Qian Du^b^*, Jiayang Liu^a^, Haiyan Tai^a^, Yinghan Chai^a^, Xiaoqiong Tan^c^, Bing Li^a^, Lirong Tan^a^, Ying Cao ^a#^，Tingting Zhu^a#^

^a^ GuiZhou University Medical College, Guiyang 550025, Guizhou Province, China.

^b^ Department of Endoscopy and Digestive System, Guizhou Provincial People’s Hospital, Guiyang, Guizhou 550002, China.

^c^ Department of Respiratory and Critical Care Medicine, The Affiliated Hospital of Kunming University of Science and Technology, Kunming, Yunnan, China.

* - author shared co-first authorship

# - co-corresponding authors

**Correspondence to:**

Professor Tingting Zhu, E‑mail: ttzhu@gzu.edu.cn

Professor Ying Cao, E‑mail: caoy@gzu.edu.cn

GuiZhou University Medical College, Guiyang 550025, Guizhou Province, China.

Xiahui Road, Huaxi District, Guiyang, Guizhou 550025, P.R. China;

**Supplementary Figures 1.**

**
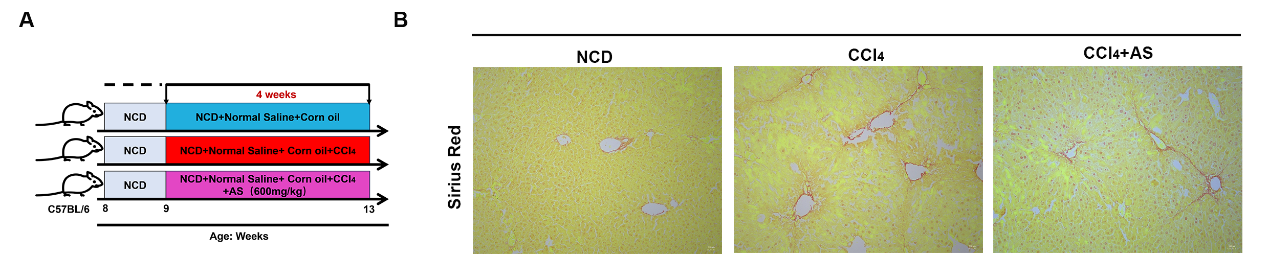
**

**Supplementary Figure 1.**. AS slows down carbon tetrachloride-induced liver fibrosis. (A) Mice were randomly allocated into groups and subjected to different treatments. Control, Model, AS with n = 6 per group. CCl₄ was administered at 0.5 mL/kg by intraperitoneal injection twice weekly, followed by oral gavage of acetylshikonin (AS = 600mg/kg) daily for 4 weeks. (B) Representative photomicrographs of liver sections stained with Sirius red to visualize collagen deposition.

**Supplementary Figures 2.**

**
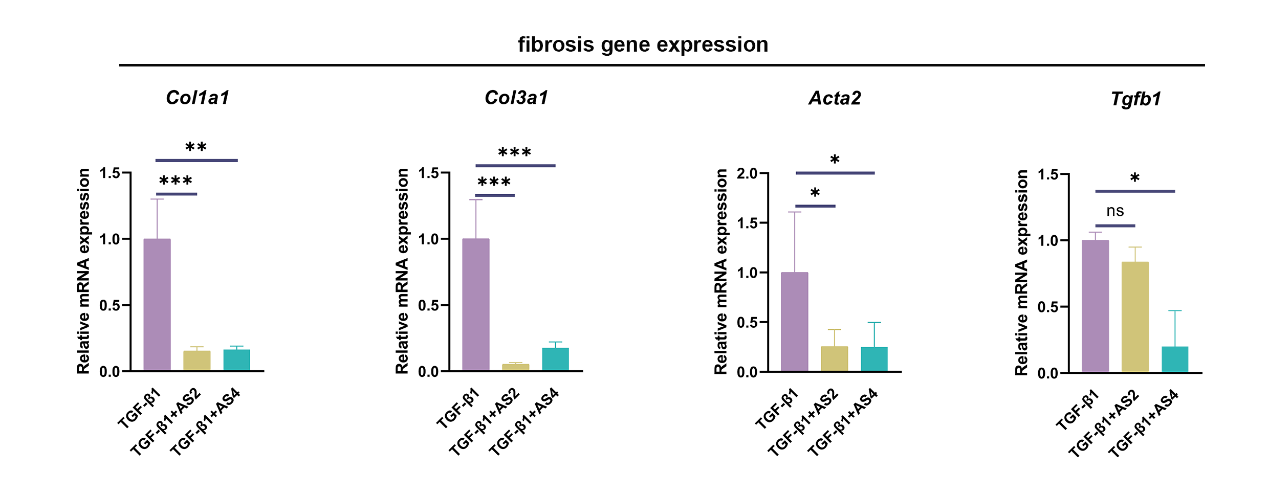
**

**Supplementary Figure 2.** qPCR analysis of the fibrosis gene expression levels of *Col1a1, Col3a1, Acta2* and *Tgfb1* after AS treatment in TGF-β1-induced LX2 cells. Data are presented as mean ± SD (n=3); **p*<0.05, ***p*<0.01, ****p*<0.001.

**Supplementary Figures 3.**

**
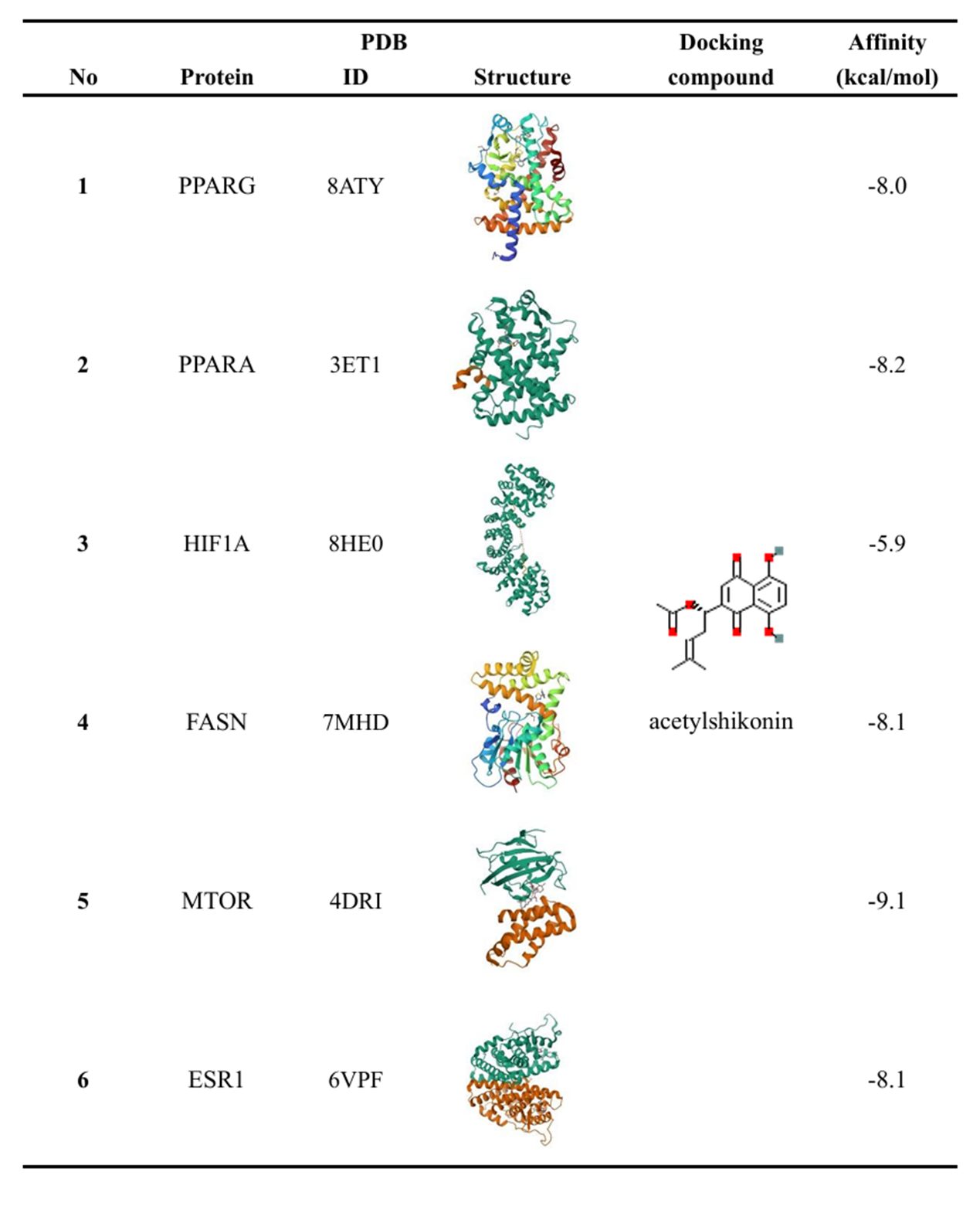
**

**Supplementary Figure3.** Molecular docking information of acetylshikonin and core targets.

**Supplementary Figures 4.**

**
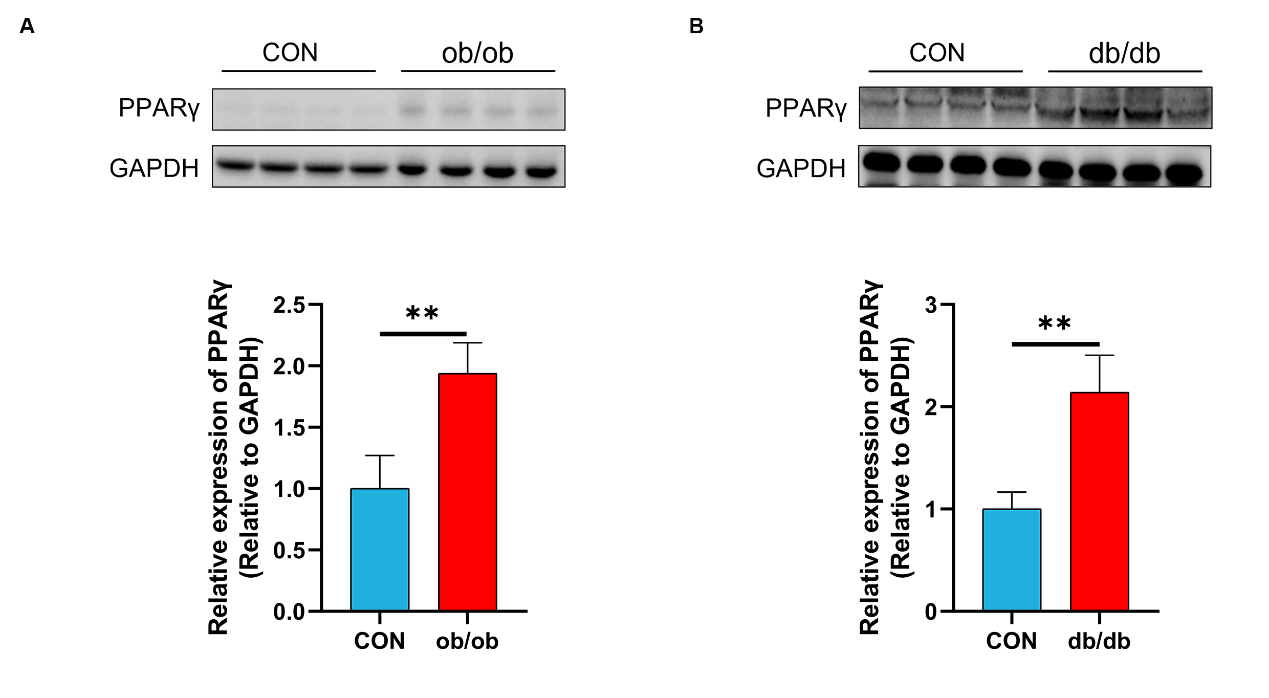
**

**Supplementary figure 4.** Western blot analysis of PPARG protein expression in ob/ob and db/db mice. All quantitative data are shown as mean ± SD; **p*<0.05, ***p*<0.01, ****p*<0.001.
