# Supplementary material for: Acetylshikonin mitigates diet-induced MASLD by targeting PPARγ-mediated metabolic dysfunction
Source: Front Pharmacol. 2026 Feb 23;17:1735481. doi: 10.3389/fphar.2026.1735481 (PMC12968015; doi:10.3389/fphar.2026.1735481)
Supplement: Supplementary file 4 [file Table1.docx]

| **Real-time PCR primer sequences** | | | |
| --- | --- | --- | --- |
| **Gene** | **Species** | **Forward Primer** | **Reverse Primer** |
| PPARγ | Mouse | AGCATTTCTGCTCCACACTAT | GGTTCTACTTTGATCGCACTTTG |
| PPARα | Mouse | GGGCAAGAGAATCCACGAAG | GTTGTTGCTGGTCTTTCCCG |
| CD36 | Mouse | GTTATTGGTGCAGTCCTGGC | ACGTCATCTGGGTTTTGCAC |
| HMGCR | Mouse | TCTGGCAGTCAGTGGGAACTATT | CCTCGTCCTTCGATCCAATTT |
| SREBP1C | Mouse | CTGGTGAGTGGAGGGACCAT | GACCGGTAGCGCTTCTCAAT |
| SREBP2 | Mouse | GACATCATCTGTCGGTGGTG | GGGCTCTCTGTCACTTCCAG |
| CCL2 | Mouse | TTAAAAACCTGGATCGGAACCAA | GCATTAGCTTCAGATTACGGGT |
| MCP1 | Mouse | CAGCAGGTGTCCCAAAGAAG | TTCCGATCCAGGTTTTTAAT |
| IL10 | Mouse | GCTCTTACTGACTGGCATGAG | CGCAGCTCTAGGAGCATGTG |
| IL8 | Mouse | CAGAGCTGCGCTGTCAGTG | TTCAGGGTCAAGGCAAACTT |
| FN1 | Mouse | CTTTGGCAGTGGTCATTTCAG | TGGTAGGTCTTCCCATCGTCA |
| TNFα | Mouse | CAGAAAGCATGATCCGCGAC | GGTCTGGGCCATAGAACTGA |
| COL1A1 | Mouse | CGGAGCAGACGGGAGTTTC | GATGTCTTCTTGGCCATGCG |
| ACTA1 | Mouse | AGACCTTCAACGTGCCTG | CGTCCCCAGAATCCAACAC |
| GAPDH | Mouse | GGCACAGTCAAGGCTGAGAATG | ATGGTGGTGAAGACGCCAGTA |
| COL1A1 | Human | GAGGGCCAAGACGAAGACATC | CAGATCACGTCATCGCACAAC |
| COL3A1 | Human | TTGAAGGAGGATGTTCCCATCT | ACAGACACATATTTGGCATGGTT |
| ACTA2 | Human | AGAAGAGTTACGAGTTGCCTGATGG | GCTGTTGTAGGTGGTTTCATGGATG |
| TGF-β1 | Human | CTCCCGTGGCTTCTAGTGC | GCCTTAGTTTGGACAGGATCTG |
| GAPDH | Human | ATGCCTCCTGCACCACCAACT | ATGGCATGGACTGTGGTCATGAGT |
